# Machine Learning Classification of Articular Cartilage Integrity Using Near Infrared Spectroscopy

**DOI:** 10.1007/s12195-020-00612-5

**Published:** 2020-03-09

**Authors:** Isaac O. Afara, Jaakko K. Sarin, Simo Ojanen, Mikko A. J. Finnilä, Walter Herzog, Simo Saarakkala, Rami K. Korhonen, Juha Töyräs

**Affiliations:** 1 Department of Applied Physics, University of Eastern Finland, Kuopio, Finland; 2 Diagnostic Imaging Centre, Kuopio University Hospital, Kuopio, Finland; 3 Research Unit of Medical Imaging, Physics and Technology, Faculty of Medicine, University of Oulu, Oulu, Finland; 4 Human Performance Laboratory, Faculty of Kinesiology, University of Calgary, Calgary, Canada; 5 Department of Diagnostic Radiology, Oulu University Hospital, Oulu, Finland; 6 School of Information Technology and Electrical Engineering, The University of Queensland, Brisbane, Australia

**Keywords:** Osteoarthritis, Cartilage, Near infrared spectroscopy, Machine learning, Deep learning, Classification

## Abstract

**Introduction:**

Assessment of cartilage integrity during arthroscopy is limited by the subjective visual nature of the technique. To address this shortcoming in diagnostic evaluation of articular cartilage, near infrared spectroscopy (NIRS) has been proposed. In this study, we evaluated the capacity of NIRS, combined with machine learning techniques, to classify cartilage integrity.

**Methods:**

Rabbit (*n* = 14) knee joints with artificial injury, induced *via* unilateral anterior cruciate ligament transection (ACLT), and the corresponding contra-lateral (CL) joints, including joints from separate non-operated control (CNTRL) animals (*n* = 8), were used. After sacrifice, NIR spectra (1000–2500 nm) were acquired from different anatomical locations of the joints (*n*_*TOTAL*_ = 313: *n*_CNTRL_ = 111, *n*_CL_ = 97, *n*_ACLT_ = 105). Machine and deep learning methods (support vector machines–SVM, logistic regression–LR, and deep neural networks–DNN) were then used to develop models for classifying the samples based solely on their NIR spectra.

**Results:**

The results show that the model based on SVM is optimal of distinguishing between ACLT and CNTRL samples (ROC_AUC = 0.93, kappa = 0.86), LR is capable of distinguishing between CL and CNTRL samples (ROC_AUC = 0.91, kappa = 0.81), while DNN is optimal for discriminating between the different classes (multi-class classification, kappa = 0.48).

**Conclusion:**

We show that NIR spectroscopy, when combined with machine learning techniques, is capable of holistic assessment of cartilage integrity, with potential for accurately distinguishing between healthy and diseased cartilage.

**Electronic supplementary material:**

The online version of this article (10.1007/s12195-020-00612-5) contains supplementary material, which is available to authorized users.

## Introduction

Osteoarthritis (OA) is a disabling musculoskeletal condition affecting a significant proportion of the world’s population. OA is mainly associated with damage and erosion of articular cartilage, the functional soft tissue surrounding the ends of articulating bones. OA could be triggered by aging (idiopathic OA) or injury to cartilage and other joint connective tissues, leading to cartilage lesions, which without intervention may degenerate to post-traumatic OA (PTOA). Surgical intervention in the repair of cartilage injury is currently conducted *via* arthroscopy, which allows visual inspection of the intra-articular joint space for assessment of lesion size, depth and severity. However, diagnosis of the extent of cartilage injuries during arthroscopy is limited by the subjective visual nature of the technique,^31^ decreasing the chances of successful intervention. Thus, methods with potential for sensitive and reliable arthroscopic assessment of cartilage integrity would be highly desirable.

There has been growing interest in utilizing fast optical methods, particularly near infrared spectroscopy (NIRS), for the evaluation of cartilage conditions.^1^,^2^,^5^^–^7,19,22,27,28,33 NIRS is capable of characterizing cartilage physical^5^,^27^ and functional^6^,^27^ properties, and composition.^1^,^22^,^28^ Furthermore, NIRS has been proposed for assessing^4^,^23^,^32^ cartilage integrity and monitoring the progression of OA using an animal model,^3^ which provides *in vivo* and *ex vivo* data during methods development and optimization. Thus, the method has the potential to overcome the limitations of current conventional arthroscopy and enable quantitative diagnosis of cartilage condition during repair surgery.

During degeneration, complex and often simultaneous micro- and macroscopic changes occur within the matrix of articular cartilage, which consists mainly of collagen, proteoglycans (PG) and water. For example, the early stages of OA are characterized by increased water content, loss of PG, and degeneration of the superficial collagen network, resulting in altered mechanical responses. Since NIRS is sensitive to specific molecular species containing CH, NH, OH and SH bonds, which constitute the fundamental structure of cartilage composition, it can detect changes in the tissue during degeneration.^3^ Thus, the NIR spectrum of cartilage contains information on its physico-chemical, structural, and functional characteristics.

Analysis of NIR spectra for assessment of cartilage integrity has so far relied on traditional multivariate linear methods, particularly partial least squares regression (PLSR), e.g., for prediction of specific tissue properties from cartilage NIR spectrum. Only few studies have applied machine learning techniques, such as support vector machines (SVM)^3^ and neural networks (ANN),^28^,^30^ for analysis of cartilage NIR spectral data, specifically regression analysis. However, no study has applied machine learning techniques for classification of connective tissue integrity based on NIRS. Furthermore, machine learning algorithms, particularly deep learning methods such as convolutional neural networks, are gaining interests in the diagnosis of musculoskeletal disorders, with recent studies,^8^,^35^ demonstrating their capacity for diagnosis of OA from radiographic images with unprecedented accuracy.

Classification of cartilage integrity based on NIRS harnesses the capacity of the spectrum, which encodes latent and inherent properties of the cartilage matrix, to provide a holistic assessment of the tissue. For example, features in the spectrum, such as spectral peaks and shapes and shapes due to absorption and scattering associated with the composition and structure of cartilage, encode information on tissue thickness^5^ and biomechanical^27^ properties. In this study, we compared the performance of traditional machine learning techniques, including support vector machines (SVM) and logistic regression (LR), with deep learning methods, particularly deep neural networks (DNN), for classification of cartilage integrity based on NIRS. SVM is a supervised learning algorithm that uses the maximum margin principle to find a hyperplane that best separates two or multiple classes, while LR uses the logit (sigmoid) function to model the relationship between independent (predictors) and response (classes) variables based on maximum likelihood estimation. Unlike traditional analytical approach that is problem-defined, neural networks are universal approximators. They can discover features in large datasets by using the backpropagation algorithm to indicate how a model should change its internal parameters that are used to compute the representation in each layer from the representation in the previous layer.^15^ We hypothesized that machine learning techniques, which encompass both traditional (e.g., SVM and LR) and state-of-the-art (e.g., DNN) artificial intelligence techniques, can harness sample-related information embedded in the NIR spectrum for classification and holistic assessment of the tissue integrity.

## Materials and Methods

### Sample Preparation and Experimental Protocol

Unilateral anterior cruciate ligament transection (ACLT) surgery, to induce degenerative changes consistent with early stage osteoarthritis,^18^ was performed on skeletally mature female New Zealand white rabbits (*n* = 14, age = 12 months at the time of operation) under general anaesthesia. Anesthesia was induced by first delivering a pre-med sedative (SQ, Acepromazine maleate, 1 mg/kg body weight, AceVet®, Vetoquinol Inc., Lavaltrie, QC, Canada). After 30 minutes, animals were placed under deep surgical anesthesia using 5% Isoflurane (Fresenius Kabi Inc., Richmond Hill, ON, Canada) in medical oxygen (1 l/min). Anesthesia was maintained with 1 to 2% Isoflurane in medical oxygen, and monitored continuously.

**Figure 1:**
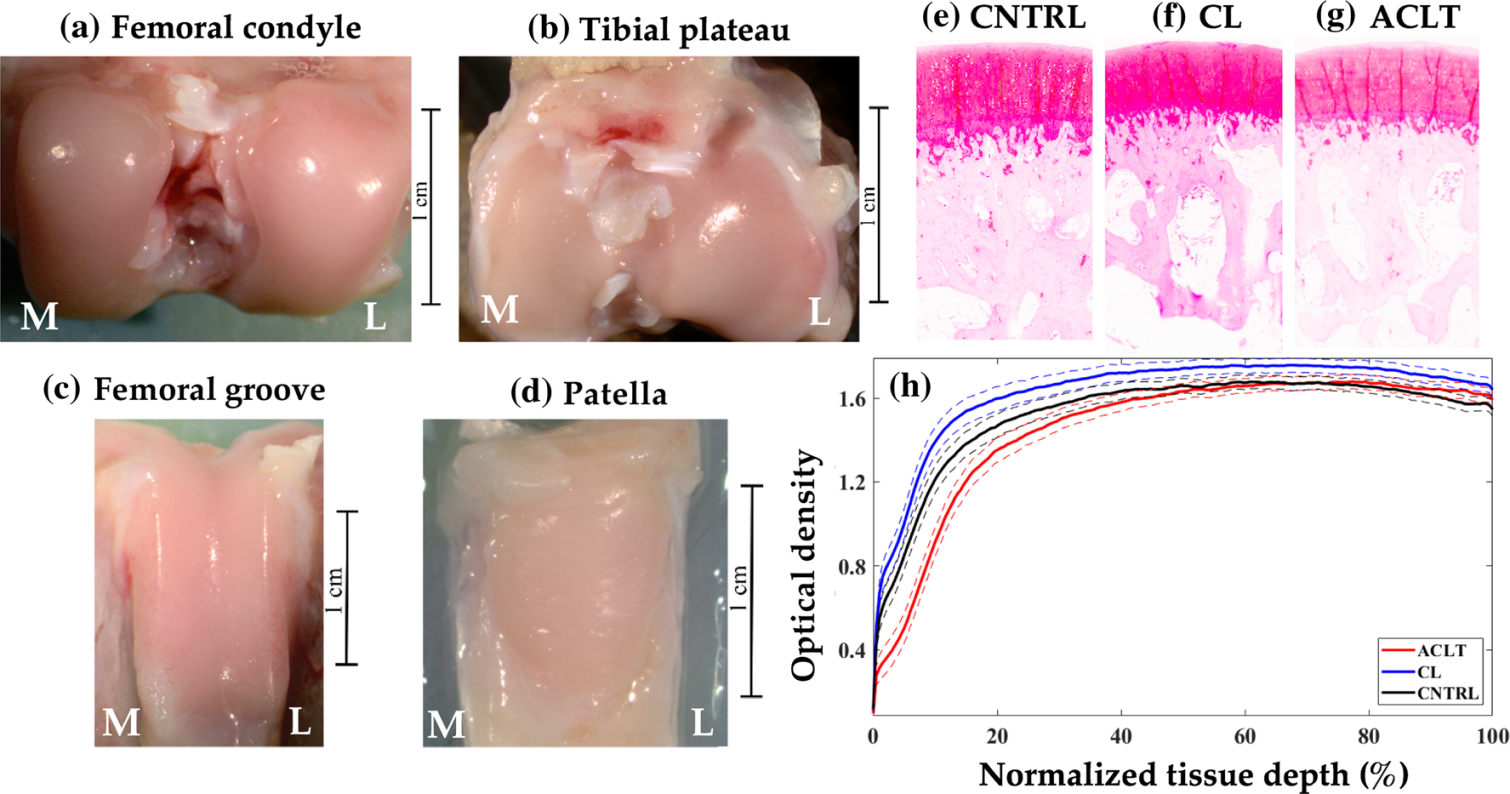
Rabbit knee joint showing anatomical locations (a–d) where spectral measurements were collected, and representative Safranin-O stained sections obtained from the medial femoral condyle of control (CNTRL, e), contra-lateral (CL, f) and anterior cruciate ligament transected (ACLT, g) joints. (h) shows the average (thick line) and 95% CI (dashed line) of proteoglycan (PG) content profile of samples from the different groups. [*M* medial, *L* lateral].

Animals were sacrificed under general anesthesia at two time points post-injury: 2 weeks (*n* = 8 animals, 16 knee joints) and 8 weeks (*n* = 6 animals, 12 knee joints). Subsequently, the experimental (ACLT) and contra-lateral (CL) knee joints were harvested from the animals after sacrifice. Control (CNTRL) knee joints (*n* = 16) were collected from age and gender-matched animals (*n* = 8) not subjected to any surgical procedure. Subsequently, osteochondral (cartilage-bone) samples were collected from patella, lateral femoral groove, the medial and lateral tibial plateaus, and the femoral condyles (Figs. 1a–1d) from all joints. Spectral measurements and histological analysis were performed on all samples, resulting in a total of 313 (*n*_CNTRL_ = 111; *n*_CL_ = 97; *n*_ACL_ = 105) measurement locations. All experiments were carried out in accordance with guidelines of the Canadian Council on Animal Care and were approved by the committee on Animal Ethics at the University of Calgary [#AC11-0035].

### NIR Spectroscopy

Diffuse reflectance NIRS of the samples was performed using an Avantes dual VIS-NIR spectrometer (AvaSpec-ULS2048L, wavelength 350–1100 nm and AvaSpec-NIR256-2.5-HSC, wavelength 1000–2500 nm, Avantes BV, Apeldoorn, The Netherlands) and light source (AvaLight HAL-(S)-mini, wavelength 360–2500 nm, Avantes BV, Netherlands). Spectral data were acquired using a custom-designed arthroscopic fibre optic probe (Avantes BV). The reusable stainless-steel fibre probe (diameter = 3.25 mm) is autoclave sterilisable (121 °C) and has a tip shaped like a traditional arthroscopic hook. The probe (window diameter = 2 mm) contains 114 optical fibres (diameter = 100 μm), with 100 emitting fibres and 14 collecting fibres (7 fibers collecting light from the sample back to each spectrometer). To ensure sample preservation and physiological conditions, each sample’s surface was covered with 0.15 M phosphate- buffered saline (PBS) solution at room temperature during spectral measurements. Although the entire probe tip (diameter = 3.2 mm) did not always cover the sample surface as a result of the curvature, the central region consisting of the receiving fibres (diameter = 1 mm) of the probe window (diameter = 2 mm)^25^ was always in full contact with the sample surface during spectral acquisition. Contact was made between the probe tip and sample surface before spectral acquisition to avoid spectral absorption from the surrounding PBS. Spectral acquisition was performed using the AvaSoft software (ver. 8.7.0, Avantes BV).

Three spectral measurements were acquired per sample (1.8 seconds/measurement), with probe realignment prior to each measurement. Each spectrum consisted of 100 co-added (averaged) scans, with the final spectrum for each sample obtained as the average of the three spectral measurements, resulting in a total of 313 spectra (*n*_CNTRL_ = 111, *n*_CL_ = 97, *n*_ACLT_ = 105). Due to spectral saturation, *i.e.*, near complete absorption of the NIR photons, in the combination region of the NIR spectral range (1900–2500 nm), data used in the analyses were restricted to 1000–1900 nm (140 variables).

### Histological Analysis and Tissue Grading

Following NIRS measurements, the samples were subjected to histological analysis for assessment of tissue integrity. Briefly, the samples were fixed in formalin, decalcified with ethylenediaminetetraacetic acid (EDTA), dehydrated in a series of graded alcohols and embedded in paraffin according to the standard protocol.^20^,^21^ Subsequently, 3 *μ*m thick sections were cut from the samples and stained with Safranin-O (Figs. 1e–1g), a cationic stain that binds stoichiometrically to negatively charged glycosaminoglycans in PG. Following staining, the sections were subjected to standard digital densitometry protocol^20^ to quantify the depth-wise PG distribution (Fig. 1h).

### Spectral Pre-processing

**Figure 2:**
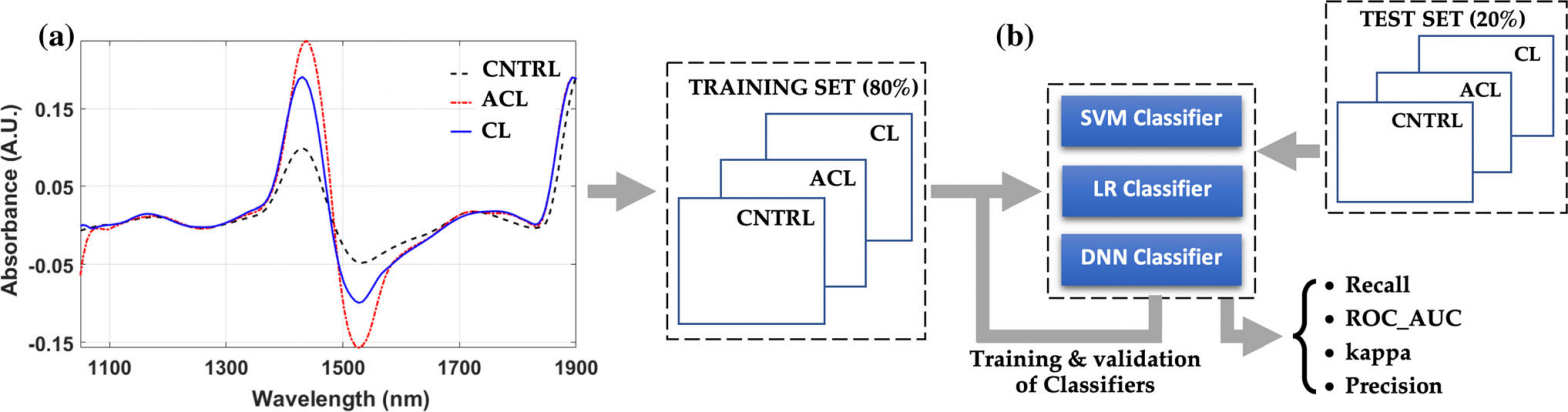
Schematic illustration of the analysis protocol showing (a) representative first derivative pre-processed NIR spectra and (b) protocol for training, validation, and testing of classifiers performance.

Since NIRS measurements are prone to noise due to ambient environment and stray light, pre-processing is often required to minimise the influence of noise on the outcome of spectral analysis. In this study, spectral pre-processing was performed using Savitzky–Golay filter with different combinations of parameters. Filter window sizes of 9 (≈ 57 nm) and 11 (≈ 69.7 nm), polynomial order (2 and 3), and three different derivative orders: 0 (spectral smoothing), 1 (1st derivative) and 2 (2nd derivative, Fig. 2a), were applied to generate different pre-processed spectra used in machine learning analyses for classification of cartilage integrity.

### Classification Models and Parameter Tuning

For assessment of cartilage integrity, classification models (Fig. 2b) were developed using traditional machine learning methods, including support vector machines (SVM) and logistic regression (LR), and deep learning methods, specifically deep neural networks (DNN). Three different classifiers were developed, including two binary classifiers: ‘*classifier 1*’ for distinguishing between CNTRL and ACLT samples, and ‘*classifier 2*’ for differentiating between CNTRL and CL samples from their NIR spectra. The third classifier (‘*classifier 3*’) was aimed at multi-class classification of cartilage integrity, i.e., differentiating between CNTRL, CL and ACLT, akin to classifying between healthy, moderate and advanced cartilage degeneration, respectively. Prior to analysis, the joints were split by animals into training (≈ 80%) and test (≈ 20%) sets to avoid biased estimators. Furthermore, in order to account for site-specific  variations in cartilage properties, the sample splitting was done based on animals so that the training and test set data included samples from the different anatomical locations. Sample number distribution for the different classification problems are: *n*_classifier_1_training_ = 173 (*n*_ACLT_ = 84, *n*_CNTRL_ = 89), *n*_classifier_2_training_ = 165 (*n*_CL_ = 76, *n*_CNTRL_ = 89), *n*_classifier_3_training_ = 249 (*n*_ACLT_ = 84, *n*_CL_ = 76, *n*_CNTRL_ = 89), *n*_classifier_1_test_ = *n*_classifier_2_test_ = 43, *n*_classifier_3_test_ = 64.

Since SVM and LR consist of multiple hyperparameters that alter the decision boundary for classification, and affect a model’s performance on independent data, it is essential to find the combination of hyperparameters that optimizes model performance and generalization. To achieve optimal performance, a ‘fit’ and ‘score’ method was employed using the GridSearchCV algorithm.^9^ In this algorithm, a model is first fitted on the training data using a classifier (e.g., based on SVM or LR) with a set of hyperparameter values. The resulting model is then validated and scored using a specified metric, *e.g.*, mean absolute error (MAE) was used in this study. The process is repeated with different combinations of hyperparameter values, allowing an exhaustive cross-validated search over a user-defined grid of classifier hyperparameters, where the best classifier hyperparameters are those that yield the lowest error score.

Two different DNN architectures were developed for the deep learning-based classifiers, with the optimal architecture determined after preliminary tests. The DNN architecture for the binary classification models (*classifier 1* and *classifier 2*) consisted of 4 fully-connected (dense) layers, consisting of 70, 70, 35 and 14 neurons. The architecture for multi-class classification (*classifier 3*) consisted of 5 fully-connected layers, consisting of 140, 140, 70, 35 and 14 neurons. A dropout layer^34^ (rate = 0.4) was inserted between the last hidden layer and the output layer to minimize overfitting. Based on preliminary model assessment, hyperbolic tangent (‘*tanh’*) function was optimal for neuron activation in all layers, except the output layer, where ‘*softmax’* activation was used because the networks are designed for classification. During training, the training set was further (randomly) divided into training (90%) and validation (10%) sets. During each training step, validation metrics (accuracy and loss) were calculated and used to tune the model’s hyperparameters (weights and biases). Optimal network hyperparameters were achieved using the Adam optimization algorithm in all networks, with ‘*binary_crossentropy*’ and ‘*categorical_crossentropy*’ loss functions in the binary and multi-class networks, respectively. Model training was halted when no improvement in validation metrics are observed after 20 consecutive epochs.

Classifier performance was assessed in terms of precision, recall, f1-score, kappa and area under receiver operator characteristic (ROC_AUC). It is worth noting that in binary classification, recall of the positive class (CNTRL) is the ‘sensitivity’ of the classifier; while recall of the negative class (ACLT and CL) is the ‘specificity’ of the classifier. Machine learning analysis was performed using *Scikit-Learn*^24^ (ver. 0.20.1) package in Python (ver. 3.6). Deep learning analysis was performed using Keras deep learning library^12^ (ver. 2.2.4), with TensorFlow^13^ (ver. 1.12.0) backend, in Python (ver. 3.6).

## Results

**Figure 3:**
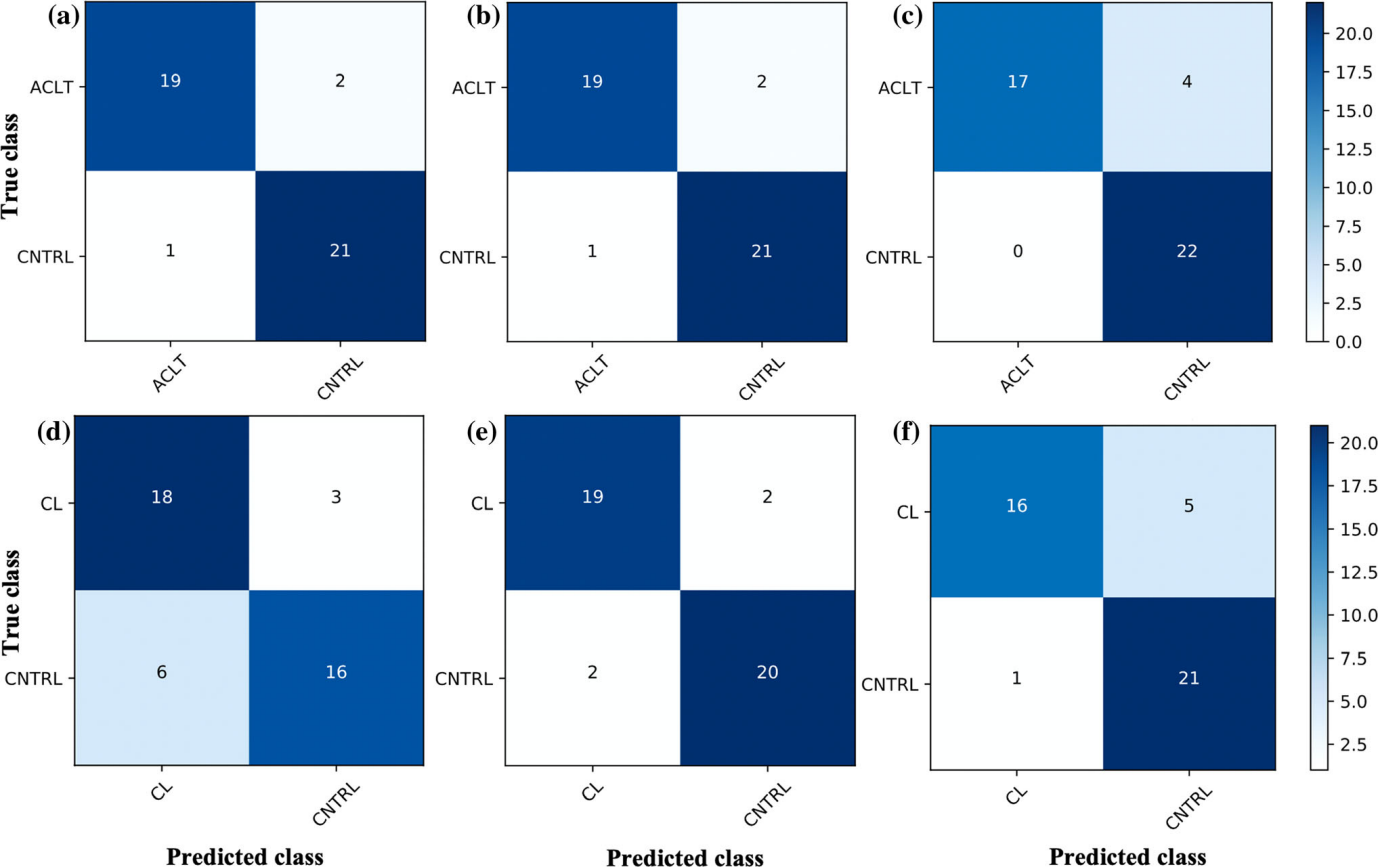
Confusion matrix showing prediction performance of the binary classification models. Performance of classifier 1 and classifier 2 models based on SVM (a: kernel = linear and C=10; d: C = 1000, gamma = 0.001, kernel = rbf), LR (b: regularization penalty = *l*2, C = 1000; e: regularization penalty = *l*1, C = 1000) and DNN (c and f) for predicting cartilage integrity in the independent test set, respectively. Optimal spectral preprocessing was based on Savitzky–Golay filtering with window size of 9, polynomial order of 2 and no derivative.

Histological analysis shows significant decrease in depth-wise PG content at the superficial and middle zones of the ACLT group compared to the CNTRL group, and throughout the cartilage depth compared to the CL group (Fig. 1h). Spectral pre-processing with a filter window of 9, polynomial order of 2, and no derivative (i.e., only spectral smoothing) of the NIR spectra yielded the best performance for both binary and multi-class cases, possibly due to noise and loss of spectral fidelity after derivative pre-processing. For distinguishing between ACLT and CNTRL samples (classifier 1), SVM (optimal parameters: C = 10, kernel = linear) and LR (optimal parameters: C = 1000, regularization = *l*2) were optimal, with similar levels of performance (Table 1, Figs. 3a and 3b). However, in differentiating between CL and CNTRL samples (classifier 2), the LR model (optimal parameters: C = 1000, regularization = *l*1) was optimal (Fig. 3e), with SVM yielding the worst result (Fig. 3d). Although DNN performed slightly poorer than the traditional methods in binary classification, it was better at classifying CNTRL samples (Figs. 3c and 3f).

**Table 1:** Performance metrics of the best classifiers for assessing cartilage integrity based on NIRS: best classifier 1 for differentiating between ACLT and CNTRL is based on SVM; best classifier 2 for differentiating between CL and CNTRL is based on LR; and best classifier 3 for multi-class classification is based on DNN.

	Precision	Recall	f1-score	ROC_AUC	kappa
Classifier 1					
ACLT	0.95	0.90	0.93	0.93	0.86
CNTRL	0.91	0.95	0.93		
Classifier 2					
CL	0.90	0.90	0.90	0.91	0.81
CNTRL	0.91	0.91	0.91		
Classifier 3					
ACLT	0.58	0.52	0.55	–	0.48
CL	0.63	0.57	0.60		
CNTRL	0.73	0.86	0.79

**Figure 4:**
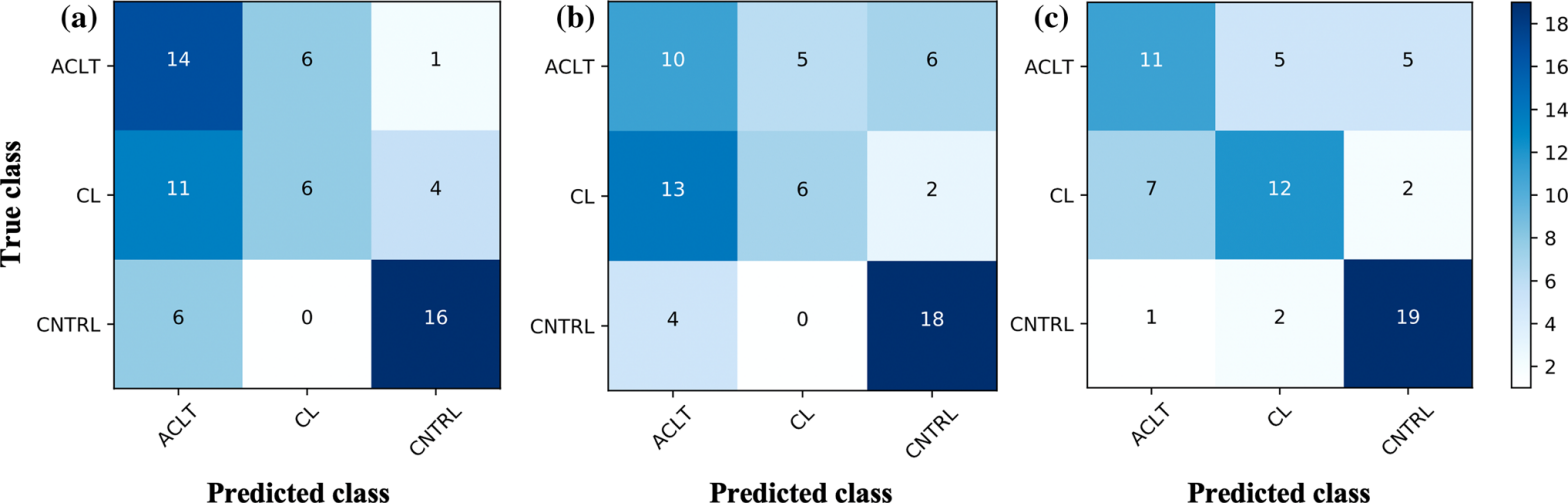
Confusion matrix of the best multi-class classification models based on SVM (a, kernel = linear, and C=100), LR (b: regularization penalty = *l*1, C = 1000) and DNN (c) The optimal spectral preprocessing was based on Savitzky–Golay filtering with window size of 9, polynomial order of 2 and no derivative.

For multi-class classification, the model based on DNN outperformed SVM and LR (Fig. 4c), albeit with poorer performance compared to the binary classifiers. The results also show that more samples were misclassified by the SVM and LR multi-class models (Figs. 4a and 4b), with most of the misclassifications occurring with the ACLT samples being classified as CL, suggesting that these classifiers have poor sensitivity in distinguishing between the NIR spectra of ACLT and CL samples.

## Discussion

We demonstrate the capacity of NIRS, combined with machine learning methods, to support a holistic assessment of cartilage integrity *via* classification analysis. We quantified the potential of NIRS to distinguish between cartilage of control and experimental joints, simulating differences between healthy and degenerated cartilage. Although NIRS has been extensively applied for predicting cartilage biochemical^1^,^22^,^30^ and structural^28^ properties, which are the basis of its function, these properties would need to be measured for each location independently because of the site-specific variation in cartilage properties. Furthermore, the functional properties of articular cartilage, alone, are not reliable indicators of tissue integrity. This is supported by the observation of Brown *et al*.,^10^ who showed significant overlap in the stiffness of normal and degenerated cartilage due to site-specific variations in articular cartilage structure, functional and material properties. More so, besides regression analysis for prediction of cartilage properties,^28^,^30^ it is worth noting that no study has applied machine learning techniques for classification of cartilage integrity based on non-destructive spectroscopic methods like NIR spectroscopy, which has immense diagnostic potential.

While the exact mechanism of cartilage degradation and pathophysiological process in OA remains unclear, a complex interplay between factors including genetics, environment, metabolism and biochemistry has been proposed.^16^ During degeneration, matrix-degrading enzymes are overexpressed, resulting in degradation of the matrix network on a molecular level. This leads to decrease in the size of matrix molecules and eventual loss of cartilage extra-cellular matrix (collagen and PGs), together with a concomitant increase in tissue water content. This degenerative process initiates a proliferative chondrocyte response with synthesis of increased quantities of extra-cellular matrix macromolecules. Even in samples with marked histological alterations, some chondrocytes can be observed to display strong anabolic activities, often characterized by cell clusters (cell cloning) surrounded by newly synthesized matrix molecules. However, as the disease progresses, the chondrocytes are not able to keep up their repair activity and the reparative attempts are outpaced by cartilage degradation^26^ resulting in complete loss of the cartilage tissue. Probing these changes by taking advantage of the interaction of light with the structure and composition of cartilage enables identification of integrity-related features in the spectra, *e.g.*, increased OH peaks (~ 1450 and ~ 1900 nm)^22^ from increased water content, for tissue assessment. Thus, adaptation of machine learning-based approaches takes advantage of the relevant integrity-related features in the NIR spectrum for classification of tissue health, which may be difficult to capture *via* single traditional macroscopic parameters, such as instantaneous and dynamic moduli.

Depending on the anatomical location, samples from the contra-lateral (CL) joints may exhibit slightly higher PG content compared to CNTRL samples, as opposed to the lower PG content observed in the ACLT samples (Fig. 1h). This may be due to exposure of the CL joints to abnormal loading as a result of altered gait following injury of the ACLT joint,^14^,^18^ triggering increased synthesis of PG by chondrocytes, a physiological response observed in the early stages of cartilage degeneration, and a precursor to gradual PG loss and matrix disruption.^17^ Thus, CL and ACLT joints present different manifestation of early stage cartilage matrix degeneration. As such, the main changes observed in samples with early matrix alteration are related to changes in PG content, which influences the water content and potentially the superficial collagen structure, all of which are detectable using NIRS.^1^,^2^,^22^,^30^ This result suggests that cartilage degeneration, and thus changes in its spectral features, may not follow a linear trend, and therefore may benefit from a non-linear modelling approach such as neural networks, including shallow and deep networks.

**Figure 5:**
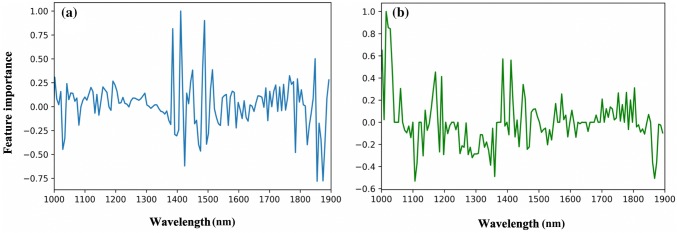
Normalized feature importance of SVM classifier 1 (a) and LR classifier 2 (b) models.

Differentiating between CNTRL and ACLT samples was trivial for all classifiers, even at this early stage of cartilage degeneration, possibly due to significant differences between the spectra of samples from both classes. Inspection of the coefficients of the optimal classifiers (*via* the feature importance plot, Fig. 5), obtained as part of the standard output from the machine learning analysis, provides insight into the variables, and thus the specific matrix components, that contribute substantially to the classification between sample classes. The features in classifier 1 (Fig. 5a) show that spectral variables related to the water peaks (1370–1550 nm and 1850–1900 nm) are the most dominant. This is indicative of the increased water content in the ACLT samples during degeneration—one of the significant and noticeable changes during cartilage degeneration.^11^

In the case of classifier 2, which is aimed at distinguishing between CNTRL and CL samples, it is unclear why LR outperformed the other methods. However, the difference in performance is not that substantial (Figs. 3d–3f). The dominant features in the LR classifier (Fig. 5b) are spread across the spectrum, suggesting that differences between the classes (CL and CNTRL) might be due to overall changes within the tissue matrix. In multi-class classification, aimed at evaluating the capacity of NIRS to differentiate between CNTRL, CL and ACLT samples, the deep learning-based model was optimal, possibly due to the capacity of neural networks to model non-linear relationships. The performance observed with the DNN model suggests that the relationship between the spectra and cartilage integrity consists of both linear and non-linear changes in cartilage components, which is consistent with the progression of cartilage degeneration.

The high misclassifications rate of CL observed in the multi-class case, particularly in the SVM and LR multi-class models, might be due to pooling of samples from the animals sacrificed at 2 and 8 weeks, potentially resulting in samples with inconsistent (location-specific) trends of tissue degeneration within the same class, since certain locations exhibit more pronounced changes at 2 weeks than at 8 weeks, and *vice versa*. Nevertheless, the deep learning model was able to classify the samples with somewhat reasonable performance (Fig. 4c). Since deep learning models require a large amount of data for training, the DNN model performance is likely to improve in all classification cases when more data are available. Furthermore, the approaches adopted in this study would need to be further validated using large animal models and human joints *ex vivo*. In a recent study, Sarin *et al*.^28^ applied shallow neural networks for predicting cartilage properties from its NIR spectrum, thus supporting the outcomes and underlying hypothesis of this study that machine learning methods may offer better approaches for classification of cartilage integrity from NIR spectra than traditional methods.

In conclusion, NIRS combined with machine learning techniques, could provide a powerful tool for classification of cartilage integrity, with the potential for accurately distinguishing between normal and early osteoarthritic cartilage. This finding, combined with recent application of NIR for estimating cartilage biomechanical properties in human cadaver knee joints,^25^,^29^ is insofar significant as it suggests that NIRS can be adapted for rapid diagnosis of cartilage integrity during knee arthroscopy, where it may be critical to correctly discriminate between healthy and degenerated cartilage prior to removal of the degenerated tissue. However, our approach needs further validation with human samples prior to clinical applications in cartilage/joint repair surgery.

## Electronic supplementary material

Below is the link to the electronic supplementary material.

Supplementary material 1 (PNG 4416 kb) Figure S1: Plot of the raw NIR spectra and different combination of spectral preprocessing based on Savitzky-Golay filter. Win = window with, der = derivative.
